# Qualitative and Quantitative Microbiological Studies of Paediatric Artemether-Lumefantrine Dry Powders and Paracetamol Syrups Obtained from Selected Drug Stores in Accra, Ghana

**DOI:** 10.1155/2019/7062016

**Published:** 2019-07-14

**Authors:** Solomon Opoku, Isaac Nyanor

**Affiliations:** ^1^Quality Control Department, Entrance Pharmaceuticals and Research Centre, No. 16 Okpoi Gonno, Spintex Road, Post Office Box CT 10805, Accra, Ghana; ^2^Sickle Pan Africa Research Consortium, Kumasi Centre for Sickle Cell Disease, Komfo Anokye Teaching Hospital, Kumasi, Ghana

## Abstract

Infants and children under five years generally have high susceptibility to pathogenic and opportunistic infections due to immaturity and inexperience of their immune responses. The lives of these young children are threatened when they consume pharmaceutical preparations of poor microbiological quality. Considering the widespread use of artemether-lumefantrine dry powder and paracetamol syrup among the general population in Ghana, there is a need to investigate the microbiological quality and safety of these paediatric pharmaceutical preparations. The study investigated the microbiological quality of 180 samples comprising 90 artemether-lumefantrine dry powders and 90 paracetamol syrups. The samples were tested for presence of specified indicator pathogens, Total Aerobic Microbial Count (TAMC), and Total Yeasts and Moulds Count (TYMC) using compendial procedures. Results from the study indicated that 16 (17.78%) of the paracetamol syrup samples showed bioburden levels above United States Pharmacopeia (USP) maximum acceptable limit, but none of the artemether-lumefantrine dry powder samples recorded microbial load above the limit of USP. Four samples of paracetamol syrup and 4 samples of artemether-lumefantrine dry powder showed presence of* P*.* aeruginosa*, whereas 5 samples of paracetamol syrup were found to be contaminated with* Salmonella* spp. Overall, 4.44% of the artemether-lumefantrine dry powders and 25.56% of the paracetamol syrups were found to be noncompliant with USP specifications for nonsterile pharmaceutical preparations for oral use. This study has revealed the existence of substandard paediatric pharmaceutical products in the Ghanaian market, hence the need for regulatory bodies to intensify monitoring and postmarketing surveillance programmes to help get rid of these products from the market.

## 1. Introduction

Malaria-related morbidity and mortality are common in children under five years from Africa where malaria transmission rate is relatively high [1]. In Ghana, malaria is major cause of mortality and morbidity, particularly among children under five years [2]. The availability of artemisinin-based compounds, obtained from the Chinese plant* Artemisia annua*, has introduced a new age in the treatment of this disease [3]. A combination of artemether and lumefantrine has been approved for the first-line treatment of infants and children with acute* Plasmodium falciparum* infections [4]. The combination is effective against strains known to be resistant to traditional antimalarials such as chloroquine and sulfadoxine-pyrimethamine and can be used to treat infections acquired in areas known to have multidrug-resistant parasites [5].

Ghana and other countries severely hit by malaria have changed their national drug policies and introduced artemether-lumefantrine as first-line therapy for uncomplicated* Plasmodium falciparum* malaria [6]. The change in policies has resulted in the proliferation of artemether-lumefantrine in the Ghanaian market and made the drug one of the artemisinin-based combinations that are commonly prescribed and used in Ghana [7].

Paracetamol syrup is an over-the-counter drug [8] used for management of fever and mild-to-moderate pain in infants and children [9]. It is one of the most widespread and most commonly used analgesic and antipyretic drugs in the world [10]. In the Ashanti region of Ghana, paracetamol syrup was found to be the most prevalent (88.8%) analgesic medicine [11]. In the treatment of fever associated with malaria, the Ministry of Health of Ghana recommends that children under five years having axillary temperature of ≥ 37.5°C or feeling very hot to touch on examination be given antipyretic, preferably paracetamol [12].

Due to the widespread use of artemether-lumefantrine dry powder and paracetamol syrup among the general population in Ghana, the microbiological safety of these drugs remains an important public health concern. Paediatric syrups and dry powders for suspension are formulated for oral administration in children under five years of age because tablets and capsules cannot be easily or conveniently administered to them [13]. These syrups and dry powders are nonsterile pharmaceutical preparations that contain sugars and other organic products and easily become substrates for pathogenic and nonpathogenic microorganisms [14]. The incidence of microbial contamination of pharmaceutical preparations has been well documented [15] with reports on recovery of pathogenic microorganisms such as* Escherichia coli*,* Staphylococcus aureus*,* Pseudomonas aeruginosa*, and* Candida albicans *from pharmaceutical preparations [16, 17]. Drug-borne infections due to consumption of these contaminated pharmaceutical products have also been reported globally [18] with descriptions of clinical risks that are attributable to consumption of these pharmaceutical preparations [19].

Infants and children under five years generally have increased susceptibility to pathogenic and opportunistic infections due to immaturity and inexperience of their immune responses [20]. The lives of these young children are threatened when they consume pharmaceutical preparations that are contaminated with pathogens or have bioburden levels out of specifications because the immune systems of these children are poorly developed [21]. Against this backdrop, it is essential to critically investigate the microbiological quality and safety of the pharmaceutical products that are produced specially for this vulnerable group. Accordingly, the aim of this study was to do qualitative and quantitative microbiological assessment of artemether-lumefantrine dry powders for oral suspension and paracetamol syrups randomly sampled from selected drug stores in Accra, Ghana.

## 2. Materials and Methods

### 2.1. Collection and Physical Examination of Samples

A list of all licensed pharmacy shops and over-the-counter medicine outlets (OTCMOs) operating in Accra was obtained from the Pharmacy Council, Ghana. Shops were selected at random using systematic random sampling technique with a sampling interval of thirty-four. A total of 57 pharmacy shops and 33 OTCMOs were included in the study. One hundred and eighty samples comprising 90 artemether-lumefantrine dry powders and 90 paracetamol syrups were purchased from the selected shops and outlets and studied. At each of the selected pharmacy shops and OTCMOs, a sample of artemether-lumefantrine dry powder and a sample of paracetamol syrup were purchased. In total, 9 different brands of the products were obtained for the study. Ten samples per product for each of the 9 brands were selected for the study. The primary packaging materials for 79 of the sampled artemether-lumefantrine dry powders were polyethylene terephthalate (PET) bottles while 11 samples were packaged in sachets. Each of the 90 paracetamol syrups samples was packaged in amber glass bottle. Each sample was taken through physical examination of appearance, odour, manufacturing date, expiry date, and brand as a means of ascertaining physical quality and originality of the product. The packaging material was also inspected for leakage and crack.

### 2.2. Work Area

All sample preparations and analyses were conducted in an ISO 8/Class 100,000 room and under positive pressure ISO 5/Class 100 horizontal laminar flow air that has passed through a High Efficiency Particulate Air (HEPA) filter. The laminar air flow (LAF) bench (Guangzhou KLC Cleantech Company Limited, Guangzhou City, China) used had efficiency of 99.99% and the capacity to filter microorganisms and particulates 0.3 micron in size. Test activities were carried out in an LAF hood to protect items and test preparations from ambient contamination [22].

### 2.3. Sterility, Growth Promotion, and Indicative and Inhibitory Properties of the Culture Media

All culture media used in this study were prepared from dehydrated media obtained from HiMedia Laboratories Private Limited, Mumbai, India. The prepared media were confirmed for sterility, growth promotion, and indicative and inhibitory properties in accordance with United States Pharmacopeia 37 [23].

### 2.4. Sample Preparation

Sample preparation was done as per the United States Pharmacopeia 37 [23]. A 1 in 10 dilution of the test product was done by aseptically diluting 10g of or 10mL of the product in sterilized Phosphate Buffer solution pH 7.2. Further dilutions, where required, were prepared with the same diluent.

### 2.5. Method Suitability Verification

The ability of tests to detect microorganisms in the presence of the test products was established. Method suitability verification was done using American Type Culture Collection (ATCC) in accordance with United States Pharmacopeia 37 [23]. Method suitability was confirmed for all microbial enumeration tests and tests for specified microorganisms carried out in this study.

### 2.6. Microbial Enumeration Tests

The pour plate method [23] was used in determining the Total Aerobic Microbial Count (TAMC) and the Total Combined Yeasts and Moulds Count (TYMC) in the artemether-lumefantrine dry powder and paracetamol syrup samples. One milliliter of the prepared sample was aseptically transferred from the prepared solution/suspension into each of four sterilized Petri dishes 9cm in diameter. To each of two of the Petri dishes, 20mL of sterilized Soybean-Casein Digest Agar (cooled approximately up to 45°C) was added for TAMC, and to the remaining two Petri dishes, 20mL of sterilized Sabouraud Dextrose Agar (cooled approximately up to 45°C) was added for TYMC. The contents of each Petri dish were mixed by gently swirling the plate to proper mixing of sample and the medium and allowed to solidify. Negative control was simultaneously performed by using 1 mL of the sterilized diluent instead of sample for each medium. Plates of Soybean-Casein Digest Agar were incubated at 35°C for 5 days for TAMC and plates of Sabouraud Dextrose Agar at 25°C for 5 days for TYMC in inverted position. After incubation, plates corresponding to a given dilution and showing the highest number of colonies less than 250 for TAMC and 50 for TYMC were selected. The arithmetic mean per culture medium of the count was taken and the number of colony-forming units (cfu) per gram or per milliliter of product was calculated.

### 2.7. Tests for Specified Microorganisms

Tests for the presence of* Escherichia coli*,* Salmonella *spp.,* Pseudomonas aeruginosa*, and* Staphylococcus aureus *in the artemether-lumefantrine dry powder and paracetamol syrup samples were conducted as per the procedure described by the United States Pharmacopeia 37 [23].

#### 2.7.1. Escherichia coli

Sample was prepared as described in Section 2.4 above and 10mL (quantity corresponding to 1g or 1mL) was used to inoculate 100mL of Soybean-Casein Digest Broth and incubated at 35°C for 24 hours. After incubation, the broth bottle was shaken for the content to mix well and 1mL of the Soybean-Casein Digest Broth transferred to 100mL of MacConkey Broth and incubated at 42°C for 24 hours. After incubation, the MacConkey broth tube was shaken and a loopful streaked on a plate of MacConkey Agar using a sterilized inoculating loop. The MacConkey Agar plates were incubated at 35°C for 24 hours in inverted position. Growth of colonies on the MacConkey Agar plates indicated the possible presence of* E*.* coli *[23]. Isolates were identified using Gram stain and biochemical tests and confirmed with API 20E (bioMérieux, Marcy l'Etoile, France) identification system [24]. The biochemical tests used for the identification of the isolates comprised oxidase test, citrate utilization test, urease test, indole test, and Kligler iron agar test [25].

#### 2.7.2. Salmonella spp.

Sample was prepared as described in Section 2.4 above and 100 mL (quantity corresponding to 10g or 10 mL) was used to inoculate 500mL of Soybean-Casein Digest Broth and incubated at 35°C for 24 hours. After incubation, the broth bottle was shaken for the content to mix well and 0.1mL of the Soybean-Casein Digest Broth transferred to 10mL of Rappaport Vassiliadis Salmonella Enrichment (RVSE) Broth and incubated at 35°C for 24 hours. After incubation, the RVSE Broth tube was shaken and a loopful streaked on a plate of Xylose Lysine Deoxycholate (XLD) Agar using a sterilized inoculating loop. The XLD Agar plates were incubated at 35°C for 24 hours in inverted position. The growth of well-developed red colonies, with or without black centres, indicated the possible presence of* Salmonella *spp. This was identified using Gram stain and biochemical tests and confirmed with API 20E (bioMérieux, Marcy l'Etoile, France) identification system [24]. The biochemical tests used for the identification of the isolates comprised oxidase test, citrate utilization test, urease test, indole test, and Kligler iron agar test [25].

#### 2.7.3. Pseudomonas aeruginosa

Sample was prepared as described in Section 2.4 above and 10mL (quantity corresponding to 1g or 1mL) was used to inoculate 100mL of Soybean-Casein Digest Broth and incubated at 35°C for 24 hours. After incubation, the Soybean-Casein Digest Broth tube was shaken and a loopful streaked on a plate of Cetrimide Agar using a sterilized inoculating loop. The Cetrimide Agar plates were incubated at 35°C for 24 hours in inverted position. Growth of colonies on the Cetrimide Agar plates indicated the possible presence of* Pseudomonas aeruginosa *[23]. Isolates were identified using Gram stain and biochemical tests and confirmed with API 20E (bioMérieux, Marcy l'Etoile, France) identification system [24]. The biochemical tests used for the identification of the isolates comprised oxidase test, citrate utilization test, urease test, indole test, and Kligler iron agar test [25].

#### 2.7.4. Staphylococcus aureus

Sample was prepared as described in Section 2.4 above and 10mL (quantity corresponding to 1g or 1mL) was used to inoculate 100mL of Soybean-Casein Digest Broth and incubated at 35°C for 24 hours. After incubation, the Soybean-Casein Digest Broth tube was shaken and a loopful streaked on a plate of Mannitol Salt Agar using a sterilized inoculating loop. The Mannitol Salt Agar plates were incubated at 35°C for 24 hours in inverted position. The growth of yellow or white colonies surrounded by a yellow zone indicated the possible presence of* S*.* aureus *[23]. Identification of* S*.* aureus* was done using Gram stain, catalase test, and the tube coagulase test [26].

### 2.8. Data Analysis

Data was captured with Microsoft Excel, cleaned, and exported to STATA version 11.0 (StataCorp., 4905 Lakeway Drive Station, Texas 77845, USA) for statistical analysis. Descriptive analysis was performed. Frequency distribution was done to show the relationship between other variables and product type. Chi-square tests and/or Fisher's exact test where appropriate was used to test associations between product type and disease causing organisms. Finally, Wilcoxon Rank Sum test was used to establish if there was a relationship between the levels of contamination (TAMC and/or TYMC) and presence or absence of any of the 4 specified indicator pathogens (*E*.* coli*,* Salmonella* spp.,* P*.* aeruginosa*, and* S*.* aureus*).

## 3. Results

### 3.1. Physical Status of Products and Packaging Materials

Physical examination of the samples did not reveal any awkward appearance or foul odour. All the products had their brand names, manufacturing dates, and expiry dates stated and were within their shelf-life at the time of the study. There was no leakage or crack on the sachets and bottles containing the products.

### 3.2. Microbial Load

Seventy-seven (85.56%) of the artemether-lumefantrine dry powder samples showed bacterial growth while 42 (46.70%) produced yeasts and moulds. More than half (60.00%) of the paracetamol syrup samples yielded bacterial growth while 41 (45.56%) produced yeasts and moulds (Table 2). Out of the 90 artemether-lumefantrine dry powder samples studied, 22 (24.44%) yielded Total Aerobic Microbial Count (TAMC) below 10 cfu/g while 68 (75.56%) produced TAMC greater than 10 cfu/g. Fifty-eight (64.44%) of the artemether-lumefantrine dry powder samples produced Total Yeasts and Moulds Count (TYMC) below 10 cfu/g while 32 (35.56%) yielded TYMC above 10 cfu/g (Table 2). Fourteen (15.56%) of the artemether-lumefantrine dry powder samples did not show any microbial contaminants (Table 2). None of the artemether-lumefantrine dry powder samples recorded TAMC above 2000 cfu/g or TYMC above 200 cfu/g.

The proportion of paracetamol syrup samples that produced TAMC below 10 cfu/mL was 46.67% while 48 (53.33%) produced TAMC above 10 cfu/mL (Table 2). Sixty-one (67.78%) paracetamol syrup samples showed TYMC below 10 cfu/mL while 29 (32.22%) samples produced TYMC above 10 cfu/mL (Table 2). The TAMC recorded by the artemether-lumefantrine dry powder samples ranged from <10 cfu/g to 9.5 x 10^1^ cfu/g and that of the paracetamol syrup samples ranged from <10 cfu/mL to 5.15 x 10^2^ cfu/mL. The artemether-lumefantrine dry powder samples recorded TYMC between <10 cfu/g and 65 cfu/g while the paracetamol syrup samples produced TYMC ranging from <10 cfu/mL to 185 cfu/mL. Thirty-five (38.89%) of the paracetamol syrup samples did not show any microbial contaminant (Table 1). Of the paracetamol syrup samples studied, 11 (12.22%) showed TAMC above 200 cfu/mL and 16 (17.78%) recorded TYMC above 20 cfu/mL.

### 3.3. Specified Indicator Pathogens

A total of 9 (10.00%) paracetamol syrup samples were found to contain pathogenic bacteria. Five of the samples produced* Salmonella* spp. while 4 showed* Pseudomonas aeruginosa *(Table 1). Four (4.44%) of artemether-lumefantrine dry powder samples produced* Pseudomonas aeruginosa *(Table 3). Neither* E*.* coli* nor* S*.* aureus* was detected in any of the samples analysed. The presence of* Salmonella* spp. was found to be more associated with paracetamol syrup than artemether-lumefantrine dry powder (p = 0.03; Table 3). None of the four pathogens was isolated from the artemether-lumefantrine dry powders packaged in sachets. There was significant relationship between the presence of* P*.* aeruginosa *and the products packaged in PET bottles (p = 0.0062; Table 4).

## 4. Discussion

Physical examination of the products and their packaging materials did not give any physical indication of substandardness. All samples were found to be in good physical conditions and free from possible ambient contaminants. This is an indication that the manufacturers of these products adhere to the packaging requirements of the Food and Drugs Authority (FDA) Ghana.

The results obtained in this study showed that majority (85.56%) of the samples showed microbial growth. The number of contaminated samples in this study is higher than that reported earlier in a similar study [27]. This high rate of contamination may be as a result of ineffective or incorrect amounts of preservatives in these formulations [28]. Active pharmaceutical ingredients (APIs), excipients, poor aseptic handling, and unhygienic environmental condition could be the major factors for the observed microbial growths in the samples [29].

More than half (64.44%) of the artemether-lumefantrine dry powder samples failed to produce fungal growth (TYMC < 10cfu/g). Varied chemical and physical factors operating in the processing and storage of these drugs result in variation in fungal contamination levels [30]. The low fungal contamination observed suggests good processing and storage conditions of these artemether-lumefantrine dry powder samples [30]. Though some of the artemether-lumefantrine dry powder samples showed high microbial growth, none exceeded the USP maximum acceptable microbial counts (TAMC ≤ 2000 cfu/g, TYMC ≤ 200 cfu/g). These products were therefore compliant with the USP specifications with respect to bioburden levels. Majority (60.00%) of the paracetamol syrup samples studied showed microbial growth. This finding is consistent with that of previous studies, where majority (60.00%) of paracetamol syrups samples were found to be contaminated with microbes [31]. Dilute oral liquid drugs such as paracetamol syrups serve as suitable media for microbial growth [21], hence the need to employ stringent hygienic techniques in the manufacturing process to minimize contamination.

Results from this study indicated that 11 (12.22%) out of the 90 paracetamol syrup samples exceeded USP TAMC limit (≤ 200 cfu/mL) while 16 (17.78%) exceeded USP TYMC limit (≤ 20 cfu/mL). Syrups with microbial counts beyond compendial limits have been reported by various studies [32–34]. Paediatric pharmaceutical products with microbial loads greater than maximum allowable bioburden counts pose serious health risk to the young children that consume them. This is because young children have poorly developed immune systems [21] and may be prone to opportunistic infections with these microbes.

Overall, brand ‘A' products showed the least bioburden levels while brand ‘B' produced the highest level of microbial contaminants. Varied manufacturing conditions and processes for the various brands explain differences in microbial contamination levels. Different ambient conditions such as temperature and relative humidity in the different drug stores might have also contributed to the differences in the levels of contaminants in the products.

Nonsterile pharmaceutical preparations, in addition to passing bioburden tests, must also pass the tests for absence of specified indicator pathogens (*P*.* aeruginosa*,* E*.* coli*,* S*.* aureus*, and* Salmonella* spp.) before they are released for consumption [23]. In this study, 4 samples of paracetamol syrup and 4 samples of artemether-lumefantrine dry powder showed presence of* P*.* aeruginosa* whereas 5 samples of paracetamol syrup were found to be contaminated with* Salmonella* spp. These products did not comply with USP specifications for nonsterile pharmaceutical preparations for oral use. Isolation of* Salmonella* spp. was more associated with paracetamol syrup than artemether-lumefantrine dry powder (p=0.03). This observation could be attributed to the ability of* Salmonella* spp. to thrive and reproduce more abundantly in moist conditions than in dry environments [35]. Pathogen survival is affected by the surface material of primary packaging of products [36]. The obtained outcome showed that there was a strong relationship between the presence of* P*.* aeruginosa *and the products packaged in PET bottles (p=0.0062). This outcome is consistent with that obtained in a previous work where survival rates of* P*.* aeruginosa *on surfaces of plastics were higher than on the surfaces of other materials [37]. The presence of these pathogens in the samples suggests contamination from the equipment or raw material or poor hygiene of the factory staff during production [33]. The presence of an indicator pathogen in a pharmaceutical product makes the product substandard [23] and indicates the possibility of contamination with other pathogens [31]. Existence of substandard pharmaceutical drugs in the Ghanaian market has also been reported by other researchers [7, 38]. Regulatory bodies should be alert and get rid of substandard drugs from the Ghanaian market.

## 5. Conclusion

This study reveals that 4.44% of the sampled paediatric artemether-lumefantrine dry powders and 25.56% of the paracetamol syrups were noncompliant with USP specifications for nonsterile pharmaceutical preparations for oral use. Existence of paediatric pharmaceutical products of poor microbiological quality in the Ghanaian market is of great public health concern considering the fact that children who take these medications naturally have weaker immunological system and are vulnerable to infections. Regulatory bodies should therefore intensify monitoring programmes and postmarketing surveillance and help enforce Good Manufacturing Practices and stricter marketing procedures to help maintain and improve quality of paediatric pharmaceutical products on the Ghanaian market.

## Figures and Tables

**Table 1:** Proportion of products stratified by dosage forms, packaging materials, sources, and pathogens.

Variable	Product Type	
	Artemether-lumefantrine dry powder, N=90	Paracetamol syrup, N=90
Dosage form		
Dry Powder for Oral Suspension	90	0
Syrup	0	90

Primary packaging material		
Amber Glass Bottle	0	90
PET Bottle	79	0
Sachet	11	0

Source of product		
OTCMO	33	33
Pharmacy Shop	57	57

Indicator Pathogens		
*E. coli*		
Detected	0	0
Absent	90	90
*Salmonella spp.*		
Detected	0	5
Absent	90	85
*P. aeruginosa *		
Detected	4	4
Absent	86	86
*S. aureus*		
Detected	0	0
Absent	90	90

**Table 2:** Results of the microbiological analyses of artemether-lumefantrine dry powder and paracetamol syrup.

Brand	Sample No.	Artemether-lumefantrine Dry Powder						Paracetamol Syrup					
		Microbial Count		Pathogens				Microbial Count		Pathogens			
		TAMC	TYMC	*E*. *coli*	*Salmonella* spp.	*P*. *aeruginosa*	*S*. *aureus*	TAMC	TYMC	*E*. *coli*	*Salmonella* spp.	*P*. *aeruginosa*	*S*. *aureus*
		(cfu/g)	(cfu/g)					(cfu/mL)	(cfu/mL)				
A	1	2.0 x 10^1^	< 10	ND	ND	ND	ND	< 10	< 10	ND	ND	ND	ND
	2	< 10	< 10	ND	ND	ND	ND	< 10	< 10	ND	ND	ND	ND
	3	< 10	< 10	ND	ND	ND	ND	< 10	< 10	ND	ND	ND	ND
	4	< 10	< 10	ND	ND	ND	ND	< 10	< 10	ND	ND	ND	ND
	5	< 10	< 10	ND	ND	ND	ND	2.0 x 10^1^	< 10	ND	ND	ND	ND
	6	1.5 x 10^1^	< 10	ND	ND	ND	ND	< 10	< 10	ND	ND	ND	ND
	7	1.0 x 10^1^	1.5 x 10^1^	ND	ND	ND	ND	< 10	< 10	ND	ND	ND	ND
	8	1.0 x 10^1^	< 10	ND	ND	ND	ND	< 10	1.0 x 10^1^	ND	ND	ND	ND
	9	< 10	< 10	ND	ND	ND	ND	< 10	< 10	ND	ND	ND	ND
	10	1.0 x 10^1^	< 10	ND	ND	ND	ND	< 10	< 10	ND	ND	ND	ND

B	1	7.5 x 10^1^	< 10	ND	ND	ND	ND	4.3 x 10^2^	1.25 x 10^2^	ND	ND	ND	ND
	2	3.0 x 10^1^	2.5 x 10^1^	ND	ND	ND	ND	4.15 x 10^2^	9.5 x 10^1^	ND	ND	ND	ND
	3	2.5 x 10^1^	< 10	ND	ND	ND	ND	2.7 x 10^2^	1.15 x 10^2^	ND	ND	ND	ND
	4	4.0 x 10^1^	5.5 x 10^1^	ND	ND	Present	ND	2.85 x 10^2^	8.5 x 10^1^	ND	Present	ND	ND
	5	6.0 x 10^1^	< 10	ND	ND	ND	ND	< 10	< 10	ND	ND	ND	ND
	6	1.5 x 10^1^	< 10	ND	ND	ND	ND	5.05 x 10^2^	8.5 x 10^1^	ND	ND	ND	ND
	7	8.0 x 10^1^	< 10	ND	ND	Present	ND	4.36 x 10^2^	8.85 x 10^2^	ND	ND	ND	ND
	8	2.0 x 10^1^	6.5 x 10^1^	ND	ND	Present	ND	3.85 x 10^2^	7.5 x 10^1^	ND	ND	ND	ND
	9	3.0 x 10^1^	5.5 x 10^1^	ND	ND	ND	ND	2.0 x 10^1^	< 10	ND	ND	ND	ND
	10	7.5 x 10^1^	2.0 x 10^1^	ND	ND	ND	ND	1.0 x 10^1^	< 10	ND	ND	ND	ND

C	1	< 10	< 10	ND	ND	ND	ND	2.5 x 10^1^	1.5 x 10^1^	ND	ND	ND	ND
	2	< 10	< 10	ND	ND	ND	ND	< 10	< 10	ND	ND	Present	ND
	3	1.0 x 10^1^	< 10	ND	ND	ND	ND	< 10	< 10	ND	ND	ND	ND
	4	1.0 x 10^1^	< 10	ND	ND	ND	ND	1.5 x 10^1^	< 10	ND	ND	ND	ND
	5	< 10	< 10	ND	ND	ND	ND	1.9 x 10^2^	1.1 x 10^2^	ND	ND	ND	ND
	6	< 10	< 10	ND	ND	ND	ND	2.5 x 10^1^	1.5 x 10^1^	ND	ND	ND	ND
	7	1.5 x 10^1^	< 10	ND	ND	ND	ND	1.0 x 10^1^	< 10	ND	ND	ND	ND
	8	< 10	1.0 x 10^1^	ND	ND	ND	ND	< 10	< 10	ND	ND	ND	ND
	9	2.5 x 10^1^	< 10	ND	ND	ND	ND	< 10	< 10	ND	ND	ND	ND
	10	< 10	< 10	ND	ND	ND	ND	< 10	< 10	ND	ND	ND	ND

D	1	2.5 x 10^1^	< 10	ND	ND	ND	ND	1.4 x 10^2^	1.25 x 10^2^	ND	ND	ND	ND
	2	1.5 x 10^1^	1.5 x 10^1^	ND	ND	ND	ND	3.5 x 10^1^	1.5 x 10^1^	ND	ND	ND	ND
	3	1.5 x 10^1^	1.0 x 10^1^	ND	ND	ND	ND	3.5 x 10^1^	< 10	ND	ND	ND	ND
	4	1.5 x 10^1^	< 10	ND	ND	ND	ND	2.0 x 10^1^	< 10	ND	ND	ND	ND
	5	1.5 x 10^1^	2.5 x 10^1^	ND	ND	ND	ND	< 10	< 10	ND	ND	ND	ND
	6	4.0 x 10^1^	1.0 x 10^1^	ND	ND	ND	ND	< 10	< 10	ND	ND	ND	ND
	7	< 10	< 10	ND	ND	ND	ND	1.0 x 10^1^	< 10	ND	ND	ND	ND
	8	2.0 x 10^1^	2.5 x 10^1^	ND	ND	ND	ND	< 10	< 10	ND	ND	ND	ND
	9	1.0 x 10^1^	< 10	ND	ND	ND	ND	2.7 x 10^2^	3.0 x 10^1^	ND	ND	ND	ND
	10	1.0 x 10^1^	< 10	ND	ND	ND	ND	< 10	< 10	ND	ND	ND	ND

E	1	5.5 x 10^1^	1.5 x 10^1^	ND	ND	ND	ND	9.5 x 10^1^	3.0 x 10^1^	ND	ND	ND	ND
	2	4.0 x 10^1^	< 10	ND	ND	ND	ND	< 10	< 10	ND	ND	ND	ND
	3	1.5 x 10^1^	< 10	ND	ND	ND	ND	2.5 x 10^2^	4.0 x 10^1^	ND	ND	ND	ND
	4	2.0 x 10^1^	2.5 x 10^1^	ND	ND	ND	ND	2.0 x 10^1^	1.0 x 10^1^	ND	ND	ND	ND
	5	1.0 x 10^1^	< 10	ND	ND	ND	ND	9.5 x 10^1^	1.5 x 10^1^	ND	ND	ND	ND
	6	2.0 x 10^1^	< 10	ND	ND	ND	ND	1.0 x 10^1^	< 10	ND	ND	ND	ND
	7	< 10	2.0 x 10^1^	ND	ND	ND	ND	< 10	< 10	ND	ND	ND	ND
	8	5.5 x 10^1^	< 10	ND	ND	ND	ND	< 10	< 10	ND	ND	ND	ND
	9	< 10	2.5 x 10^1^	ND	ND	ND	ND	9.5 x 10^1^	1.5 x 10^1^	ND	ND	ND	ND
	10	1.5 x 10^1^	3.5 x 10^1^	ND	ND	ND	ND	2.5 x 10^1^	< 10	ND	ND	ND	ND

F	1	1.0 x 10^1^	< 10	ND	ND	ND	ND	1.65 x 10^2^	< 10	ND	ND	ND	ND
	2	1.0 x 10^1^	< 10	ND	ND	ND	ND	< 10	< 10	ND	ND	ND	ND
	3	2.0 x 10^1^	< 10	ND	ND	ND	ND	3.0 x 10^1^	1.5 x 10^1^	ND	ND	ND	ND
	4	9.5 x 10^1^	< 10	ND	ND	Present	ND	< 10	< 10	ND	Present	ND	ND
	5	< 10	1.0 x 10^1^	ND	ND	ND	ND	1.0 x 10^1^	< 10	ND	ND	ND	ND
	6	< 10	< 10	ND	ND	ND	ND	1.0 x 10^1^	< 10	ND	ND	ND	ND
	7	3.5 x 10^1^	1.0 x 10^1^	ND	ND	ND	ND	< 10	< 10	ND	ND	ND	ND
	8	3.5 x 10^1^	< 10	ND	ND	ND	ND	< 10	< 10	ND	ND	ND	ND
	9	1.0 x 10^1^	< 10	ND	ND	ND	ND	5.15 x 10^2^	1.85 x 10^2^	ND	ND	Present	ND
	10	1.5 x 10^1^	< 10	ND	ND	ND	ND	< 10	< 10	ND	ND	ND	ND

G	1	1.0 x 10^1^	< 10	ND	ND	ND	ND	1.75 x 10^2^	1.10 x 10^2^	ND	ND	ND	ND
	2	1.5 x 10^1^	< 10	ND	ND	ND	ND	1.0 x 10^1^	1.0 x 10^1^	ND	ND	ND	ND
	3	1.5 x 10^1^	< 10	ND	ND	ND	ND	< 10	< 10	ND	ND	ND	ND
	4	< 10	2.0 x 10^1^	ND	ND	ND	ND	2.5 x 10^1^	< 10	ND	ND	ND	ND
	5	2.0 x 10^1^	< 10	ND	ND	ND	ND	2.65 x 10^2^	< 10	ND	ND	ND	ND
	6	2.0 x 10^1^	< 10	ND	ND	ND	ND	2.0 x 10^1^	1.0 x 10^1^	ND	ND	ND	ND
	7	< 10	4.0 x 10^1^	ND	ND	ND	ND	< 10	< 10	ND	ND	ND	ND
	8	1.5 x 10^1^	1.5 x 10^1^	ND	ND	ND	ND	< 10	< 10	ND	ND	ND	ND
	9	1.5 x 10^1^	2.5 x 10^1^	ND	ND	ND	ND	2.0 x 10^1^	< 10	ND	ND	ND	ND
	10	1.5 x 10^1^	< 10	ND	ND	ND	ND	1.5 x 10^1^	< 10	ND	ND	ND	ND

H	1	1.5 x 10^1^	2.0 x 10^1^	ND	ND	ND	ND	< 10	< 10	ND	ND	ND	ND
	2	1.5 x 10^1^	3.5 x 10^1^	ND	ND	ND	ND	< 10	< 10	ND	ND	ND	ND
	3	2.5 x 10^1^	4.0 x 10^1^	ND	ND	ND	ND	< 10	< 10	ND	Present	Present	ND
	4	1.0 x 10^1^	< 10	ND	ND	ND	ND	< 10	< 10	ND	ND	ND	ND
	5	1.0 x 10^1^	< 10	ND	ND	ND	ND	< 10	< 10	ND	Present	ND	ND
	6	3.0 x 10^1^	2.5 x 10^1^	ND	ND	ND	ND	9.5 x 10^1^	3.0 x 10^1^	ND	ND	ND	ND
	7	< 10	< 10	ND	ND	ND	ND	1.60 x 10^2^	1.05 x 10^2^	ND	ND	ND	ND
	8	2.0 x 10^1^	< 10	ND	ND	ND	ND	1.0 x 10^1^	1.0 x 10^1^	ND	ND	ND	ND
	9	2.0 x 10^1^	< 10	ND	ND	ND	ND	< 10	< 10	ND	ND	Present	ND
	10	1.0 x 10^1^	3.0 x 10^1^	ND	ND	ND	ND	< 10	< 10	ND	ND	ND	ND

I	1	1.5 x 10^1^	< 10	ND	ND	ND	ND	< 10	< 10	ND	ND	ND	ND
	2	1.5 x 10^1^	< 10	ND	ND	ND	ND	< 10	< 10	ND	ND	ND	ND
	3	2.0 x 10^1^	< 10	ND	ND	ND	ND	< 10	< 10	ND	ND	ND	ND
	4	2.5 x 10^1^	2.0 x 10^1^	ND	ND	ND	ND	1.5 x 10^1^	< 10	ND	ND	ND	ND
	5	1.5 x 10^1^	1.0 x 10^1^	ND	ND	ND	ND	3.5 x 10^1^	1.5 x 10^1^	ND	ND	ND	ND
	6	< 10	< 10	ND	ND	ND	ND	3.0 x 10^1^	1.5 x 10^1^	ND	ND	ND	ND
	7	< 10	1.0 x 10^1^	ND	ND	ND	ND	< 10	< 10	ND	ND	ND	ND
	8	< 10	< 10	ND	ND	ND	ND	3.5 x 10^1^	< 10	ND	ND	ND	ND
	9	5.0 x 10^1^	< 10	ND	ND	ND	ND	< 10	< 10	ND	Present	ND	ND
	10	1.5 x 10^1^	1.5 x 10^1^	ND	ND	ND	ND	1.0 x 10^1^	< 10	ND	ND	ND	ND

TAMC: Total Aerobic Microbial Count, TYMC: Total Yeasts and Moulds Count, cfu: colony-forming units, ND: not detected.

**Table 3:** Associations between product type and presence of pathogens.

Pathogen	Product Type		Total	p-value
	Artemether-lumefantrine Dry Powder	Paracetamol Syrup		
*Salmonella* spp.				
Absent	90	85	175	*0.03∗*
Detected	0	5	5	
Total	90	90	180	

*P. aeruginosa*				
Absent	86	86	172	0.64
Detected	4	4	8	
Total	90	90	180	

*S. aureus*				
Absent	90	90	180	-
Detected	0	0	0	
Total	90	90	180	

*E. coli*				
Absent	90	90	180	-
Detected	0	0	0	
Total	90	90	180	

**∗** *Fisher's test.* P< 0.05 means significant; P> 0.05 means not significant.

**Table 4:** Relationship between the levels of contamination (TAMC) and presence of *P*. *aeruginosa* in the products packaged in amber glass bottles and PET bottles.

TAMC/Material Type/Disease-causing organisms	Observation	Wilcoxon Rank sum test	Expected frequency	p value
Material Type (Amber Glass Bottle)				
*P*. *aeruginosa*				
Absent	86	3949.5	3913	0.4597
Detected	4	145.5	182	
Combined	90	4095	4095	
Material Type (PET Bottle)				
*P*. *aeruginosa*				
Absent	75	2879	3000	*0.0062*
Detected	4	281	160	
Combined	79	3160	3160	

P< 0.05 means significant; P> 0.05 means not significant.

## Data Availability

The continuous and qualitative data used to support the findings of this study are available from the corresponding author upon request.
